# Pregnancy-Associated Spontaneous Coronary Artery Dissection: A Report of the iSCAD Registry

**DOI:** 10.1001/jamacardio.2026.1009

**Published:** 2026-03-29

**Authors:** Agnes Koczo, Anna Grodzinsky, Esther S. H. Kim, Sahar Naderi, Gerald Chi, Daniella Kadian-Dodov, Heather L. Gornik, Bryan Wells, Angela Taylor, Lori Tam, Connie Hess, Jennifer Lewey, Stanislav Henkin, James Orford, Gretchen Wells, Rina Mauricio, Kathryn J. Lindley, C. Michael Gibson, Katherine K. Leon, Malissa J. Wood, Jennifer A. Sumner, Nandita S. Scott

**Affiliations:** 1 University of Pittsburgh Medical Center, Pittsburgh, Pennsylvania; 2 Saint Luke’s Mid America Heart Institute/University of Missouri-Kansas City, Kansas City; 3 Wake Forest University School of Medicine, Atrium Health, Sanger Heart & Vascular Institute, Charlotte, North Carolina; 4 Kaiser Permanente Northern California, San Francisco, California; 5 PERFUSE Study Group, Beth Israel Deaconess Medical Center, Harvard Medical School, Boston, Massachusetts; 6 Zena and Michael A. Wiener Cardiovascular Institute, Icahn School of Medicine at Mount Sinai, New York, New York; 7 University Hospitals Harrington Heart & Vascular Institute, Cleveland, Ohio; 8 Emory University School of Medicine, Atlanta, Georgia; 9 University of Virginia Medical Center, Charlottesville; 10 Providence Heart Institute, Portland, Oregon; 11 University of Colorado School of Medicine, Aurora; 12 University of Pennsylvania Perelman School of Medicine, Philadelphia; 13 Dartmouth-Hitchcock Medical Center, Lebanon, New Hampshire; 14 Intermountain Heart Institute, Intermountain Medical Center, Salt Lake City, Utah; 15 Gill Heart and Vascular Institute, University of Kentucky School of Medicine, Lexington; 16 University of Texas Southwestern Medical Center, Dallas; 17 Vanderbilt University Medical Center, Nashville, Tennessee; 18 SCAD Alliance, Alexandria, Virginia; 19 University of California, Los Angeles; 20 Mass General Brigham Heart Vascular Institute, Women’s Heart Health Program, Boston, Massachusetts

## Abstract

**Importance:**

Spontaneous coronary artery dissection (SCAD) is an increasingly recognized cause of myocardial infarction predominantly affecting women. There is a paucity of reproductive health data surrounding pregnancy-associated SCAD (P-SCAD).

**Objective:**

To examine detailed reproductive variables as well as demographics, psychosocial, and SCAD-event factors among women with a history of at least 1 prior pregnancy in a large, multicenter registry (iSCAD Registry).

**Design, Setting, and Participants:**

This cohort study compared data of women with P-SCAD vs women with non–pregnancy-associated SCAD (NP-SCAD) between the years 2019 and 2024 and rates of certain reproductive health features among the general reproductive-aged population in US states. Differences were analyzed using the Kruskal-Wallis test for continuous variables and χ^2^ test for categorical variables.

**Exposures:**

Pregnancy and SCAD.

**Main Outcomes and Measures:**

Patient survey-based clinical and reproductive history variables were collected and corroborated with investigator-extracted clinical and imaging data, including detailed characterization of the SCAD event.

**Results:**

Among 907 women (median [IQR] age at enrollment, 52.0 [45.1-59.4] years; median [IQR] age at first SCAD event, 49.2 [42.3-57.1] years) with SCAD and 1 or more pregnancies, 98 had P-SCAD with median (IQR) age of 36.7 (33.7-39.1) years at time of SCAD event. Those with P-SCAD had a lower prevalence of fibromuscular dysplasia (27 of 86 [31%] vs 309 of 681 [45%]; *P* = .01) but similar rates of extracoronary abnormalities. Greater use of assisted reproductive technology (ART; 25 of 97 [26%] vs 98 of 804 [12%]), greater multigravida with more than 5 gestations (13 of 98 [13%] vs 55 of 809 [7%]), and preeclampsia (24 of 98 [25%] vs 101 of 809 [13%]; *P* = .001) were reported among women with P-SCAD. Those with P-SCAD had a more severe SCAD phenotype including higher incidence of STEMI (16 of 86 [18.6%] vs 40 of 733 [5.5%]; *P* < .001), multivessel segment involvement (22 of 70 [31%] vs 101 of 588 [17%]; *P* = .004), and left ventricular ejection fraction (LVEF) lower than 40% (4 of 15 [27%] vs 6 of 105 [7%]; *P* = .006). They also experienced less LVEF recovery by 1-year follow-up. The majority (661 of 887 [75%]) of both groups were primarily medically managed.

**Conclusions and Relevance:**

From this large, multisite registry, results reveal that women with P-SCAD had greater median ages at gestation, greater history of ART use, and greater instances of multigravida and preeclampsia than women with NP-SCAD and the general reproductive-aged US population. With high percentages of vascular imaging among participants, women with P-SCAD had less fibromuscular dysplasia but similar rates of extracoronary abnormalities including dissection and aneurysms as women with NP-SCAD. In this contemporary cohort, women with P-SCAD continue to represent a higher-risk phenotype with predominantly conservative management; however, they had less LVEF recovery.

## Key Points

**Question:**

Do women with pregnancy-associated spontaneous coronary artery dissection (P-SCAD) have different reproductive features and SCAD-associated outcomes as compared with women with SCAD not associated with pregnancy (NP-SCAD)?

**Findings:**

In this cohort study including 907 women with SCAD, women with P-SCAD had significantly less fibromuscular dysplasia but similar rates of other vascular abnormalities, and similar severity of SCAD lesions but more severe acute coronary syndrome presentation and worse in-hospital major adverse cardiac events. Differences included greater adverse pregnancy outcomes and use of assisted reproductive technologies among patients with P-SCAD.

**Meaning:**

Results suggest that reproductive features may be informative to differential acute coronary syndrome presentation and outcomes in P-SCAD vs NP-SCAD.

## Teaser

This cohort study investigates if women with pregnancy-associated spontaneous coronary artery dissection (SCAD) have different reproductive features and SCAD-associated outcomes as compared with women with SCAD not associated with pregnancy.

## Introduction

Spontaneous coronary artery dissection (SCAD) is an increasingly recognized cause of nonatherosclerotic myocardial infarction.^1^ The etiology of SCAD is incompletely understood and likely multifactorial, relating to a complex interplay of a susceptible underlying vasculature and triggers that include emotional and physical stress, as well as hemodynamic and hormonal changes, particularly in SCAD related to pregnancy.^2,3^ Prior registry data have shown that individuals with pregnancy-associated SCAD (P-SCAD) have more severe presentations with increased prevalence of high-risk features including multivessel involvement and left ventricular systolic dysfunction.^4,5^ There are limited data on reproductive variables, such as a history of adverse pregnancy outcomes and use of assisted reproductive technology associated with P-SCAD as well as longitudinal left ventricular ejection fraction (LVEF) recovery data.^6,7^

As P-SCAD is a highly morbid condition for which there are limited data to guide pregnancy counseling, we sought to leverage a large SCAD registry to characterize in detail reproductive variables in those with P-SCAD compared with those with non–pregnancy-related SCAD (NP-SCAD). The iSCAD Registry is a multicenter, cross-sectional study network that includes enrolling sites across the US and Australia. This registry was created through the efforts of SCAD Alliance, a nonprofit advocacy organization of SCAD survivors and their families, who brought together experts in the field to build an independent data repository aimed at advancing the science of SCAD.

## Methods

Participants from the iSCAD Registry^8^ were enrolled from 20 sites with local institutional review board approval and following written participant consent throughout academic and community-based centers in the US and Australia from 2019 through 2024. For inclusion to the iSCAD Registry, participants had to be older than 18 years with a diagnosis of SCAD (not iatrogenic nor related to atherosclerosis) confirmed by coronary angiography. A subset of coronary angiograms was further adjudicated for SCAD and detailed coronary data was accessed by a centralized data center (Percutaneous Functions Under Sensitive Examination [PERFUSE] Study Group, Beth-Israel Deaconess Medical Center). All patients were enrolled prospectively. Exact inclusion criteria to the registry are included in a former article.^9^ We limited analysis for this study to participants in the registry who self-identified their gender as women, had any history of pregnancy, and completed a baseline questionnaire. Given that participants in this analysis self-reported as women and pregnancy was an inclusion requirement for enrollment, we will refer to all participants in this study as women. We recognize sex and gender are distinct concepts, with sex referring to biological attributes and gender reflecting socially and personally constructed identities, and we recognize that gender exists along a spectrum rather than as a binary concept. This study followed the Strengthening the Reporting of Observational Studies in Epidemiology (STROBE) reporting guidelines.

### Data Variables

Baseline medical history and SCAD event-related clinical data were collected by study investigators as previously described.^2,3,5,9^ Race and ethnicity were self-identified in participant-facing surveys and included the following: American Indian or Alaska Native, Asian, Black or African American, Hispanic or Latino, not Hispanic or Latino, multiracial, Native American or Other Pacific Islander, White, and other (unspecified). Detailed race and ethnicity were reported from this multisite registry with enrolling centers throughout the US. As many prior SCAD studies come from single centers, this study was a unique opportunity to capture the impact of this cardiac condition across diverse populations. Data included coronary angiography and other cardiac imaging at the index event and follow-up, medications use, and adverse events. LVEF decline was defined by LVEF less than 50% after SCAD event. LVEF recovery was defined as LVEF greater than or equal to 50% on follow-up echocardiogram within 1 year of follow-up. Serial echocardiogram timing was at the discretion of the clinical site and was not adjudicated by a central core laboratory. In-hospital major adverse cardiac events (MACE) were analyzed individually and as a composite outcome including cardiogenic shock, in-hospital death, in-hospital new cerebrovascular accident, in-hospital recurrent myocardial infarction (MI), and in-hospital cardiac transplant.

Reproductive and family history information was further informed by patient-based surveys in addition to psychosocial questionnaires. More comprehensive findings regarding psychosocial questionnaire findings, including pregnancy-related mental health data from this registry, has been included in a prior article.^3^ Specific reproductive details were also collected from patient questionnaires. To most comprehensively compare reproductive histories including obstetric variables among patients with SCAD, the dataset for analysis was limited to participants who identified as women and had at least 1 pregnancy.

P-SCAD was defined, consistent with prior published studies, as SCAD occurring during pregnancy or in the following 12 months after gestation.^10,11,12^ The comparator group was comprised of iSCAD Registry participants who identify as women and had NP-SCAD.

### Statistical Analysis

We analyzed patient-reported reproductive health features as well as clinical variables related to the index SCAD event. Differences between participants with P-SCAD and NP-SCAD were analyzed using Kruskal-Wallis test for continuous variables and χ^2^ test for categorical variables. Continuous data were summarized as median with IQR. Categorical data were summarized as proportions (%). Denominators were included if there was missing data on specific variables for the total cohort population. All statistical tests were 2-sided, and *P* values <.05 were considered statistically significant. Analyses were performed using SAS software, version 9.4 (SAS Institute).

## Results

A total of 907 women (median [IQR] age at enrollment, 52.0 [45.1-59.4] years; median [IQR] age at first SCAD event, 49.2 [42.3-57.1] years) were enrolled and had completed case report forms and a history of pregnancy at time of data analysis. Of these, 98 met inclusion criteria for P-SCAD. They were compared with 809 women who had a history of pregnancy but whose SCAD event was not pregnancy-associated (Figure).

**Figure.**
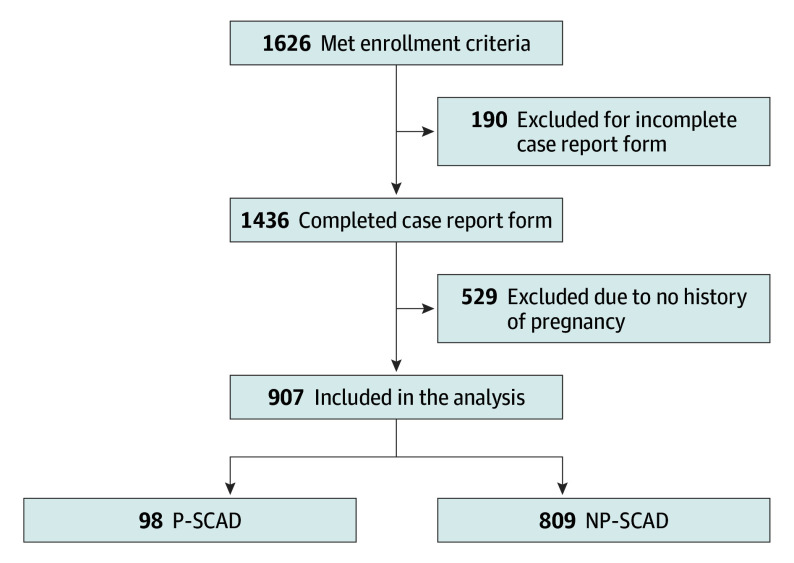
Flowchart of Participant Enrollment Flow diagram detailing enrollment schema from the comprehensive registry to inclusion of final cohorts meeting inclusion criteria for this study. Incomplete case report form denotes participant did not submit a case report form. Those who submitted (with or without any missing variables) were still included in analysis. NP-SCAD indicates non–pregnancy-associated spontaneous coronary artery dissection; P-SCAD, pregnancy-associated SCAD.

### Baseline Demographics

At the time of SCAD presentation, the median (IQR) age among participants with P-SCAD was 36.7 (33.7-39.1) years compared with the median age of 29.4 years among reproductive-aged women in the US national population.^13,14^ In this cohort, participants self-reported the following races and ethnicities: 4 American Indian or Alaska Native (0.5%) 17 Asian (2.0%), 68 Black or African American (7.8%), 46 Hispanic or Latino (5.1%), 861 not Hispanic or Latino (94.9%), 13 multiracial (1.5%), 3 Native Hawaiian or Other Pacific Islander (0.3%), 760 White (87.4%), and 5 other (0.6%). Participants with P-SCAD had fewer traditional cardiovascular risk factors, including a lower prevalence of hypertension prior to SCAD and dyslipidemia), as well as a lower prevalence of fibromuscular dysplasia (FMD; 27 of 86 [31%] vs 309 of 681 [45%]; *P* = .01). Cardiometabolic risk factors among P-SCAD participants varied as compared with national averages for similar-aged women (hypertension, 18.4% vs 12%^15^; hyperlipidemia, 4.1% vs 36.1%^15^). Among participants who underwent genetic testing, both groups demonstrated similarly low rates of inherited vasculopathies including vascular Ehlers-Danlos, Loeys-Dietz, Marfan syndrome, and autosomal-dominant polycystic kidney disease. A total of 86 of 98 participants (88%) with P-SCAD and 681 of 809 participants (84%) with NP-SCAD underwent vascular imaging screening for FMD among systemic vasculopathies. Rates of other abnormal vascular findings (aneurysm, dissection, ectasia, or tortuosity) were also comparable, although FMD was less frequently reported in the P-SCAD subgroup. In addition, the prevalence of history of anxiety or depression, migraines, and social factors such as smoking and illicit drug use were not significantly different. Notably, rates of illicit drug use with vasoactive properties (cocaine and amphetamines) were higher than population reported averaged for similar-aged women and represent a potential trigger of SCAD in both cohorts (Table 1).^16,17^

**Table 1.** Baseline Demographics Among Individuals With Pregnancy-Associated Spontaneous Coronary Artery Dissection (P-SCAD) in the iSCAD Registry vs Non–Pregnancy-Associated SCAD

Variables	No. (%)		*P* value
	P-SCAD (n = 98)	NP-SCAD (n = 809)	
Age at enrollment, median (IQR), y	38.8 (35.3-41.9)	53.1 (47.2-60.5)	<.001
Age at index SCAD, median (IQR), y	36.7 (33.7-39.1)	50.7 (44.8-58.1)	<.001
Self-identified race, No./total No. (%)			
American Indian or Alaska Native	0	4/777 (0.5)	.007
Asian	6/93 (6.5)	11/777 (1.4)	
Black or African American	12/93 (12.9)	56 of 777 (7.2)	
Multiracial	0	13/777 (1.7%)	
Native Hawaiian or Other Pacific Islander	0	3/777 (0.4%)	
White	74 (76)	695 (86)	
Other^a^	1/93 (1.1)	4/777 (0.5)	
Self-identified ethnicity, No./total No. (%)			
Hispanic or Latino	6/98 (6.1)	40/809 (4.9)	.62
Not Hispanic or Latino	92/98 (93.9)	769/809 (95.1)	
Chronic hypertension^b^	18 (18)	243 (30)	.02
Dyslipidemia	4 (4)	135 (17)	.02
Diabetes	1 (1)	21 (3)	.34
Any thyroid disease, No./total No.	12/94 (13)	117/745 (16)	.46
Migraines	53 (54)	372 (46)	.13
CVA or TIA	3 (3)	16 (2)	.48
Connective tissue conditions, No./total No.			
FMD	27/86 (31)	309/681 (45)	.01
Inherited vasculopathies^c^	1/70 (1)	4/551 (1)	.54
Aneurysm	12/86 (14)	118/681 (17)	.43
Dissection	12/86 (14)	78/681 (12)	.50
Anxiety and/or depression	25 (26)	276 (34)	.09
Tobacco, No./total No.			
Current	1/95 (1)	18/797 (2)	.47
Past	19/95 (20)	192/797 (24)	
Never	75/95 (79)	587/797 (74)	
Illicit drug use^d^	35/96 (37)	251/790 (32)	.35
Cocaine	2 (2)	49 (6)	.11
Amphetamines	0	29 (3.6)	.06
Family history			
SCAD	1 (1)	19 (2)	.40
Extracoronary dissection	3 (3)	21 (3)	.79
Hemorrhagic stroke	10 (10)	69 (9)	.58
Aneurysm	14 (14)	141 (17)	.43
Hereditary CTD	2 (2)	141 (17)	.83
FMD	2 (2)	14 (2)	.49

Abbreviations: CTD, connective tissue disorder; CVA, cerebrovascular accident; FMD, fibromuscular dysplasia; TIA, transient ischemic attack.

^a^ Other category was unspecified.

^b^ Chronic hypertension defined as hypertension prior to SCAD event.

^c^ Inherited vasculopathies include vascular Ehlers-Danlos, Loeys-Deitz, Marfan syndrome, and autosomal-dominant polycystic kidney disease.

^d^ Illicit drug use included marijuana, cocaine, amphetamine, or ecstasy.

### Reproductive History

In association with their P-SCAD event, the predominant mode of delivery was vaginal over cesarean (56 of 98 [57%] vs 36 of 98 [37%]) and 65 of 98 (66%) reported breastfeeding or pumping at the time of the SCAD event. In addition, 2 participants reported loss of pregnancy or stillbirth, and 1 participant reported preterm birth.

In regard to any pregnancy history, participants with P-SCAD reported a greater history of hypertensive disorders of pregnancy, namely very high rates of preeclampsia (24 of 98 [25%] vs 101 of 809 [13%]; *P* = .001). Among participants with P-SCAD, 17 of 98 (17%) reported any pregnancy history of preterm birth, whereas 3 of 98 (3%) had any history of pregnancy loss. Only 67% of individuals reported receiving counseling regarding future pregnancy after P-SCAD, despite the known risks associated with subsequent pregnancy.

More participants with P-SCAD were multigravida (>5 pregnancies) as compared with their counterparts with NP-SCAD (13 of 98 [13%] vs 55 of 809 [7%]; *P* = .02). A history of multifetal gestation was high in both groups as compared with the general reproductive-aged US population (approximately 32 per 1000 births)^18^; however, there was no difference between groups based on whether the SCAD was pregnancy associated or not (P-SCAD, 10 of 98 [10%] vs NP SCAD, 67 of 809 [8%]; *P* = .52) (Table 2).

**Table 2.** Pregnancy- and Reproductive-Associated Variables^a^

Reproductive and pregnancy variables	No. (%)		*P* value
	P-SCAD (n = 98)	NP-SCAD (n = 809)	
History of adverse pregnancy outcomes			
Preeclampsia	24 (25)	101 (13)	.001
Eclampsia	2 (2)	6 (1)	.19
Cervical incompetence	1 (1)	16 (2)	.51
Uterine rupture	1 (1)	4 (1)	.10
Gestational diabetes	11 (11)	54 (7)	.10
Preterm birth (<37 wk)	17 (17)	NA	NA
Loss of pregnancy or stillbirth	3 (3)	NA	NA
Pregnancy history associated with P-SCAD event			
Preterm birth	1 (1)	NA	NA
Any loss of pregnancy or stillbirth	2 (2)	NA	NA
Delivery method		NA	NA
Spontaneous vaginal delivery	56 (57)	NA	NA
Cesarean delivery	36 (37)	NA	NA
Breastfeeding and/or pumping (at time of P-SCAD event)	65 (66)	NA	NA
Reproductive history			
Age of menarche, median (IQR), y	12.5 (12.0-14.0)	13.0 (12.0-14.0)	.45
Irregular periods, No./total No.	29/84 (35)	80/210 (38)	.57
Contraception use history, No./total No.			
History of contraception use	89/98 (91)	718/808 (89)	.56
History of ART			
Any history of fertility treatment, No./total No.	25/97 (26)	98/804 (12)	<.01
Clomiphene	8 (8)	52 (6)	.51
IUI	11 (11)	40 (5)	.01
IVF	14 (14)	34 (4)	<.001
No. of IVF rounds, mean (SD)	1.9 (1.8)	2.7 (3.1)	.12
Donor egg	2 (2)	15 (1.9)	.90
Gravida			
1	12 (12)	101 (13)	.95
2	28 (29)	237 (29)	.88
3	15 (15)	224 (28)	.009
4	17 (17)	140 (17)	.99
5	15 (13)	50 (6)	.009
>5	13 (13)	55 (7)	.02
Parity			
1	21 (21)	154 (19)	.57
2	37 (38)	370 (46)	.13
3	16 (16)	161 (20)	.40
4	9 (9)	56 (7)	.41
5	4 (4)	12 (2)	.06
>5	9 (9)	21 (3)	<.001
Spontaneous or therapeutic abortions, stillbirths			
1	23 (24)	247 (31)	.15
2	16 (16)	115 (14)	.57
3	8 (8)	45 (5)	.30
4	2 (2)	11 (1)	.59
5	2 (2)	5 (1)	.13
>5	0	5 (1)	.44
Multifetal gestation			
Twins	10 (10)	67 (8)	.52
Triplets/quadruplets	0	3 (<1)	.55

Abbreviations: ART, assisted reproductive technology; IUI, intrauterine insemination; IVF, in-vitro fertilization; NA, not applicable; NP-SCAD indicates non–pregnancy-associated spontaneous coronary artery dissection; P-SCAD, pregnancy-associated SCAD.

^a^ Pregnancy and reproductive variables comparing women with P-SCAD vs NP-SCAD.

A significantly greater proportion of participants with P-SCAD reported history of any use of fertility treatment compared with those with NP-SCAD (25 of 97 [26%] vs 98 of 804 [12%]; *P* < .001), including intrauterine insemination (11 of 98 [11%] vs 40 of 809 [5%]; *P* = .01) and in vitro fertilization (14 of 98 [14%] vs 34 of 809 [4%]; *P* < .001) (Table 2).

### Mental Health

Comparisons of baseline mental health variables for participants with and without P-SCAD are presented in eTable 2 in Supplement 1. The median (IQR) time from SCAD event to registry enrollment and survey completion was 0.7 (0.2-2.6) years. A total of 14 of 95 participants (14.7%) with P-SCAD had a Generalized Anxiety Disorder 7 score cutoff consistent with probable anxiety vs 98 of 786 participants (12.5%) with NP-SCAD (*P* = .06). Similarly, 9 of 95 participants (9.5%) with P-SCAD had Patient Health Questionnaire 8 cutoffs indicating probable depression as compared with 125 of 786 participants (15.9%) with NP-SCAD (*P* = .32). Approximately 14% of both groups had sleep disturbance scores greater than 1 SD above the mean Patient-Reported Outcomes Measurement Information System T scores (*P* = .10) (eTable 3 in Supplement 1).

### Index SCAD Event

A significantly greater proportion of participants with P-SCAD presented with ST-segment elevation MI (STEMI) when compared with participants with NP-SCAD (16 of 86 [18.6%] vs 40 of 733 [5.5%]; *P* < .001). Among participants with adjudicated coronary angiograms (n = 658), those with P-SCAD (n = 70) vs NP-SCAD (n = 588) were more likely to have multivessel (22 of 70 [31%] vs 101 of 588 [17%]; *P* = .004) and multisegmented (23 of 70 [33%] vs 120 of 588 [20%]; *P* = .02) dissections. Notably, there were no cases of left main coronary artery involvement in the P-SCAD group (eTable 1 in Supplement 1).

Mean (SD) LVEF at presentation was lower in the P-SCAD group; however, the difference was not statistically significant (48.8% [12%] vs 52.9% [9%]; *P* = .34). When stratified by LVEF values, a significantly greater proportion of participants with P-SCAD had an LVEF less than 40% compared with participants with NP-SCAD (4 of 15 [27%] vs 6 of 105 [7%]; *P* = .006). A greater percentage of participants with P-SCAD also had LVEF less than 50% (3 of 15 [20%] vs 5 of 61 [8.2%]) vs NP-SCAD by echocardiogram at 1-year follow-up after a SCAD event. Among the relatively small number of patients with LVEF less than 50% after SCAD, 3 of 5 patients with P-SCAD had LVEF recovery to greater than or equal to 50% vs 8 of 19 patients with NP-SCAD by 1 year after the event. There was no significant difference in the prevalence of Takotsubo appearance on echocardiography between the groups (3 of 25 [12%] vs 42 of 220 [19%]; *P* = .39).

In the overall cohort, high-risk features were rare, with no significant differences between groups in the rates of cardiogenic shock (1 of 98 [1%] vs 1 of 809 [<1%]; *P* = .07) or cardiac arrest, whether out of hospital (1 of 98 [1%] vs 37 of 809 [5%]; *P* = .10) or in hospital (1 of 98 [1%] vs 13 of 809 [2%]; *P* = .66). However, the cohort with P-SCAD experienced a significantly higher rate of in-hospital MACE compared with the cohort with NP-SCAD (9 of 94 [10%] vs 34 of 760 [5%]; *P* = .03). This was driven by greater in-hospital recurrent myocardial infarction (8 of 95 [8.4%] vs 27 of 786 [3.4%]; *P* = .02) in the subgroup with P-SCAD (Table 3). There were no differences between groups in the use of mechanical support or in-hospital mortality.

**Table 3.** Spontaneous Coronary Artery Dissection (SCAD) Event Variables^a^^,^^b^

Variables	No./total No. (%)		*P* value
	P-SCAD (n = 98)	NP-SCAD (n = 809)	
ACS presentation			
Unstable angina	26/86 (30.2)	223/733 (30.4)	<.001
NSTEMI	44/86 (51.2)	470/733 (64.1)	
STEMI	16/86 (18.6)	40/733 (5.5)	
LVEF at presentation, mean (SD), %	48.8 (12)	52.9 (9)	.34
LVEF <50%	8/15 (53)	32/105 (31)	.08
LVEF <40%	4/15 (27)	6/105 (7)	.006
LVEF <35%	1/15 (7)	3/105 (3)	.44
LVEF <30%	1/15 (7)	3/105 (3)	.44
LVEF <50% at 1 year follow-up	3/15 (20)	5/61 (8.2)	.18
Left ventricular recovery (LVEF >50% following <50%), No./total No. (%)	3/5 (60)	8/19 (42)	.41
Takotsubo appearance	3/25 (12)	42/220 (19)	.39
High-risk features			
Cardiogenic shock	1/98 (1)	1/809 (<1)	.07
Cardiac arrest			
VF on initial ECG	1/98 (1)	13/809 (2)	.66
Presentation	1/98 (1)	37/809 (5)	.10
ICD implanted	1/14 (7)	18/100 (18)	.31
Management			
Medical management only	72/97 (74)	589/790 (75)	.94
PCI	22/97 (23)	185/792 (23)	.88
PCI-related complications			
Perforation	0/13 (0)	4/105 (4)	.47
Loss of side branch	0/13 (0)	2/105 (2)	.62
No reflow	0/13 (0)	11/105 (11)	.22
Abrupt closure	0/13 (0)	3/105 (3)	.54
Thrombus formation	0/13 (0)	3/105 (3)	.54
SCAD extension	0/13 (0)	17/105 (16)	.12
Edge dissection	0/13 (0)	10/105 (10)	.24
CABG	3/97 (3)	28/795 (4)	.83
Thrombolytics	1/90 (1)	14/739 (2)	.60
In-hospital medications			
Anticoagulation	60/81 (74)	624/715 (87)	.001
Antiplatelet therapy			
SAPT	33/98 (34)	255/777 (33)	.47
DAPT	55/98 (56)	468/777 (60)	
No antiplatelet	10/98 (10)	54/777 (7)	
β-Blockers	80/98 (82)	631/809 (78)	.41
Nitrate	28/98 (29)	313/809 (39)	.05
Complications			
Mechanical support	6/95 (6)	30/780 (4)	.25
In-hospital MACE^c^	9/94 (10)	34/760 (5)	
Cardiogenic shock	1/98 (1)	1/809 (0.1)	.03
In-hospital death	0	0	
In-hospital new CVA	0	6/768 (0.8)	
In-hospital recurrent MI	8/95 (8.4)	27/786 (3.4)	
In-hospital cardiac transplant	0	1/809 (0.1)	

Abbreviations: ACS, acute coronary syndrome; CABG, coronary artery bypass graft; CVA, cerebrovascular accident; DAPT, dual antiplatelet therapy; EKG, electrocardiogram; ICD, implantable cardioverter-defibrillator; LVEF, left ventricular ejection fraction; MACE, major adverse cardiac events; NP-SCAD, non–pregnancy-associated SCAD; NSTEMI, non–ST-elevation myocardial infarction; P-SCAD, pregnancy-associated SCAD; PCI, percutaneous coronary intervention; SAPT, single anti-platelet therapy; STEMI, ST-elevation myocardial infarction; VF, ventricular fibrillation.

^a^ SCAD event features, including presentation, management, and complications comparing women with P-SCAD vs women with NP-SCAD.

^b^ Multiple options could not selected for “Presentation” variable.

^c^ MACE were defined as composite of: recurrent myocardial infarction, new stroke, heart failure, new arrhythmia.

Most participants (661 of 887 [75%]) in both groups were managed medically, with no significant differences in the rates of percutaneous coronary intervention (PCI) (22 of 97 [23%] vs 185 of 792 [23%]; *P* = .88) or coronary artery bypass grafting (3 of 97 [3%] vs 28 of 795 [4%]; *P* = .83). Among the smaller subgroup who went on to intervention, there were no PCI-related complications in the group with P-SCAD (vessel perforation, side branch loss, no-reflow, abrupt vessel closure, thrombus formation, SCAD extension, or edge dissection).

A significantly lower proportion of participants with P-SCAD received anticoagulation during hospitalization compared with the group with NP-SCAD (60 of 81 [74%] vs 624 of 715 [87%]; *P* = .001). However, rates of in-hospital antiplatelet therapy, both single and dual antiplatelet use, as well as β-blocker and nitrate therapy, were comparable between the 2 groups in the iSCAD Registry (Table 3).

## Discussion

Pregnancy-associated SCAD is a highly morbid condition that affects individuals during a particularly vulnerable period.

### Patient Perspective

I’ve always wanted to be a mom. Having children, loving them, watching them grow… from my earliest memories, I just knew that this was what I was meant to do. To my surprise, growing and birthing tiny humans happened relatively easy for me. And now, at 35 years old, my 3 beautiful children are my world. But that world was almost ripped from me and my family in February of 2025 when I suffered a catastrophic SCAD heart attack just 6 weeks after the birth of my third baby.

It is difficult to describe what it feels like to believe you are going to die. My life until February 25, 2025, was filled with love, adventure, optimism, and confidence. On that fateful morning, I was taking my children to school along with their new baby brother. My first time out of the house on my own with all 3 in tow. At the same moment I pulled the car into the parking lot, my chest began to feel like it was ripping in half. I couldn’t breathe, my arms were numb, and a sensation of panic washed over my body. We all know where this story leads; thanks to a quick-acting school nurse, an ambulance was immediately called and I was soon admitted to the hospital with a new and personally unheard of diagnosis: a coronary dissection.

However, this is where things took a hairpin turn. Because my dissection progressed a few days later, leading to another 911 call, another elevated troponin, another heart catheterization, and ultimately cardiogenic shock and triple bypass surgery. The body that I meticulously cared for, fed whole foods, took on daily long runs, had completely betrayed me. My last memory before waking up intubated in an ICU was staring into the eyes of a physician, envisioning my children learning of my death. I whispered “I’m terrified,” and that’s all I remember.

One of my greatest life goals is to live a fulfilling and empathetic existence. Broad undertaking, I know. And I believed up until that point that I had faithfully done this. I taught about malaria in West Africa with the Peace Corps, trained for marathons, tackled a difficult master’s program, and became a mom. But my life never knew suffering until my SCAD. The hopelessness of not having any control, the physical and emotional pain, the fear; it was devastating.

Landing at a state of acceptance has not been easy. I still talk to a mental health therapist weekly and pray each night when I go to bed that I don’t die in my sleep. My greatest fear is that my baby, now 10 months old, won’t have memories of me. But when I look at the brilliant effort of my surgeons, cardiologists, nurses, the love and support from my family and community, I know that my survival wasn’t just luck. I am meant to be here. And so are the thousands of women, of mothers, who suffer SCAD worldwide.

We highlight several key findings in this contemporary study: (1) more severe SCAD phenotype in P-SCAD including more STEMI presentation, higher incidence of multivessel and multicoronary segment involvement (but no left main involvement), lower presentation LVEF (<40%), higher rates of in-hospital MACE; (2) lower rates of LVEF recovery from LVEF 50% to greater than or equal to 50% by 1 year after the event; (3) predominant conservative SCAD management in both groups; (4) less FMD among P-SCAD but similar rates of extracoronary dissections and aneurysms; (5) greater median maternal age as well as preceding adverse pregnancy outcomes as compared with US averages, namely preeclampsia and preterm delivery; (6) high use of fertility treatments before any pregnancy (26% vs 5% at mean age of 34 among the general reproductive-aged US population around 2018)^19,20^; and (7) notable mental health symptoms among patients with P-SCAD with 8.4% meeting criteria for probable posttraumatic stress disorder as well as 14.7% for anxiety. Given the peripartum status of this SCAD subgroup, it is possible these mental health scores were impacted by factors like peripartum status and pregnancy complications. Nonetheless, these findings suggest the importance of screening for and addressing poor mental health in patients after P-SCAD.

There are few studies that include detailed reproductive features comparing reproductive-aged women with and without P-SCAD. In 2017, Tweet et al^4^ similarly noted fewer traditional risk factors and less FMD among a group of 54 registry patients with P-SCAD, although the rate of vascular imaging was higher in nearly double the study population in the iSCAD cohort (85% of total participants in this study) (eTable 3 in Supplement 1). Our registry data demonstrates similar prevalence of extracoronary aneurysm and dissection while significantly less FMD comparing patients with P-SCAD vs NP-SCAD. This may reflect the impact of female sex hormone fluctuations (including lactation hormones) with known influence on vulnerable systemic vasculature and highlights the importance of head to pelvis vascular imaging in all patients with SCAD.^21^ This is further suggested by high rates of breastfeeding and/or pumping among patients with P-SCAD, in which hormones like prolactin remain high to support lactation but fluctuate based on infant demand or during breastfeeding wean. Given the timing of SCAD is often during hormonal transitions (postpartum, perimenopause), it is possible that breastfeeding may also represent a hormonal vulnerability window. As compared with other P-SCAD studies, our registry found slightly older median maternal age at time of P-SCAD presentation, compared with the median age of pregnancy in the general reproductive aged US population.^13^ In addition, we observed lower rates of anticoagulation use in the cohort with P-SCAD compared with the group with NP-SCAD (eTable 3 in Supplement 1). We hypothesize that this may be due to considerations surrounding the pregnancy and postpartum setting, including peripartum bleeding and safety of these medications during breastfeeding.^22,23^ Our study noted a greater overall history of preeclampsia (25% vs 11%) and similar assisted reproductive technology rates (28% vs 26%) among a more diverse group with P-SCAD compared with reproductive-aged women in the US population (eTable 3 in Supplement 1). The higher prevalence of preeclampsia is particularly notable given increasing evidence of systemic endothelial dysfunction that persists well beyond placental delivery. The higher prevalence of preeclampsia is particularly notable given increasing evidence of systemic endothelial dysfunction that persists well beyond placental delivery.^24^ The higher-than-average overlap between P-SCAD and preeclampsia may extend beyond the antepartum period and represents a shared, vascular susceptibility phenotype. Interestingly, we observed a higher-than-expected prevalence of multifetal gestation in both groups compared with US population data. This finding was present in both the cohort with P-SCAD and the cohort with NP-SCAD and warrants further investigation. This is an important finding among prior published series of P-SCAD in which reproductive variables were not evaluated^10,25^ (eTable 3 in Supplement 1).

Contrary to prior reports, a substantial proportion of participants with P-SCAD in this contemporary cohort (collected from 2019-2024) were treated conservatively (74% in iSCAD vs 32%-43% in prior P-SCAD studies), possibly reflecting an evolving clinical approach favoring more measured care, even within this higher-risk population with P-SCAD (eTable 3 in Supplement 1).^4,10,25,26^ Despite primarily conservative treatment, participants with P-SCAD were a higher risk group, more often with STEMI, presenting LVEF less than 40%, and greater in-hospital MACE. These worse outcomes may reflect proximity to the peripartum state in which hemodynamic stress and dynamic levels of sex hormones continue to impact vulnerable vascular, evidenced by significant rates of extracoronary aneurysm and dissection, well into the postpartum setting. Despite the physical stressors of pregnancy and known parallels between Takotsubo cardiomyopathy, rates of noting this stress cardiomyopathy were lower among patients with P-SCAD vs those with NP-SCAD. Takotsubo is most common in the general population among postmenopausal-aged women and may explain prevalence differences between the cohorts.^27^ A better understanding of factors driving worse outcomes is imperative to improving care among patients with P-SCAD. Given severity of presentation and worse outcomes associated with P-SCAD, careful discussion about subsequent pregnancies should be undertaken with every patient.

Notably, our registry included fewer cases of left main (LM) involvement than prior registry-based studies (Tweet et al^4^) as well as systematic reviews (Havekuk et al^26^). Similar to our study, Chan et al^10^ also did not find LM involvement among their cohorts with P-SCAD (eTable 3 in Supplement 1). We hypothesize that our results differ from Havekuk et al^26^ because this systematic review included many case reports/series that inherently describe severe cases. It is possible that our findings are due to excluding those with in-hospital deaths, although no in-hospital death (or deaths at follow-up) is reported in the registry-based P-SCAD study by Tweet et al^4^ despite greater LM involvement, and may argue against survivorship bias driving differences in our findings.^4^ Differences between our study and those described in eTable 3 in Supplement 1 include a large portion of our coronary angiograms that were adjudicated in detail for coronary involvement of SCAD by a centralized core laboratory with expertise in coronary angiography. In addition, our diverse patient enrollment and multisite registry with among the largest sample size of P-SCADs may represent a more modern, real-world depiction of LM involvement among patients with P-SCAD. There were no in-hospital deaths reported in the cohort with P-SCAD, which may reflect contemporary improvements in disease management for this patient population.

### Strengths and Limitations

This cohort study has several strengths. This study leveraged the large, multicenter iSCAD Registry to enhance our current understanding of P-SCAD presentation and outcomes. The registry’s broad representation of participating centers across diverse sociodemographic and geographic settings may provide a more comprehensive view of SCAD than prior single-center, primarily academic referral hospitals. This multisite design may yield a cohort that more closely approximates the true clinical spectrum of SCAD in the US. This is evidenced by a more racially diverse population compared with prior reports, with nearly one-quarter of participants who did not self-identify as White. An additional strength of this study is the inclusion of individuals with P-SCAD occurring up to 1 year postpartum or after pregnancy loss, capturing both acute postpartum presentations and later events, including those occurring after weaning from breastfeeding. Another strength is the rigorous case ascertainment, with SCAD diagnoses adjudicated by a core angiographic laboratory with expertise in coronary imaging.

We acknowledge the limitations of our analyses, including individuals’ self-report of reproductive health features as well as mental health surveys, most of which were in reference to any prior pregnancy in both groups and may have been several years before registry participation. This introduces the potential for recall bias. Further, although registry participants who submitted a case report form were included, participants may have chosen to not provide data on certain variables in the case report form and constitute missing data. However, the iSCAD Registry comprises investigator case report forms, which serve to corroborate or complete the clinical data. Greater detail on reproductive features, including duration of exposure to contraception and assisted reproductive technology as well as the temporal relationship of that exposure to the SCAD event, was not collected and will be considered in future research. Another limitation is that our acute coronary syndrome categorization was selected by sites and could not be further adjudicated. However, with the initial 658 coronary angiograms included in this study adjudicated by the core laboratory, we have core laboratory-validated details on coronary data. Given the registry nature of enrollment, it is possible that this is a lower-risk cohort and that mortality is underreported. However, no in-hospital or follow-up P-SCAD-associated deaths were reported in other studies, including those with left main involvement^10^ (eTable 3 in Supplement 1). Nonetheless, our analyses contribute to a broader understanding of reproductive health features not previously reported in a large cohort. Moreover, this study represents 1 of the largest and most diverse P-SCAD subset cohorts published to date.

## Conclusions

This cohort study contributes contemporary data to the current literature on pregnancy-associated SCAD thereby enhancing our understanding of this distinct clinical entity. In this large multisite registry, participants with P-SCAD had greater use of fertility therapies, more adverse pregnancy outcomes, and a higher incidence of parity (>5) pregnancies compared with registry participants with NP-SCAD and the general reproductive-aged US population.^12,15^ Most individuals were treated conservatively, and among those who underwent percutaneous coronary intervention, no complications were reported. Despite similar rates of extracoronary vascular abnormalities and SCAD lesion severity, those with P-SCAD had greater MACE.

Further study of reproductive, sociodemographic, and sex hormone–related factors at the time of the index SCAD event and during subsequent pregnancies is essential to clarify the more severe SCAD presentation and worse outcomes noted among patients with P-SCAD.

## Notes

Supplement 1. **eTable 1.** Adjudicated Coronary Angiograms Features **eTable 2.** Mental Health Questionnaires **eTable 3.** Comparison of P-SCAD Variables in iSCAD vs Other Registries

Supplement 2.
Data Sharing Statement.
