# Supplementary material for: Pregnancy-Associated Spontaneous Coronary Artery Dissection: A Report of the iSCAD Registry
Source: JAMA Cardiol. 2026 Mar 29;11(6):534–43. doi: 10.1001/jamacardio.2026.1009 (PMC13034164; doi:10.1001/jamacardio.2026.1009)
Supplement: Supplement 1. — eTable 1. Adjudicated Coronary Angiograms Features eTable 2. Mental Health Questionnaires eTable 3. Comparison of P-SCAD Variables in iSCAD vs Other Registries [file jamacardiol-e261009-s001.pdf]

Supplementary Online Content

Koczo A, Grodzinsky A, Kim ESH, et al. Pregnancy-associated spontaneous coronary artery dissection: a report of the iSCAD Registry. *JAMA Cardiol*. Published online March 29, 2026. doi:10.1001/jamacardio.2026.1009

eTable 1. Adjudicated Coronary Angiograms Features

eTable 2. Mental Health Questionnaires

eTable 3. Comparison of P-SCAD Variables in iSCAD vs Other Registries

This supplementary material has been provided by the authors to give readers additional information about their work.

© 2026 Koczo A et al. *JAMA Cardiology*.

eTable 1- Adjudicated Coronary Angiograms Features

Variables	P-SCAD (n=70)	NP-SCAD (n=588)	P-Values
Coronary Location			
-Left main	0 (0%)	3/588 (1%)	0.55
-LAD	47 (67%)	351 (60%)	0.23
-LCx	34 (49%)	204 (35%)	0.02
-RCA	15 (21%)	145 (25%)	0.55
SCAD type			
1	13/70 (18.6%)	66/588 (11.2%)	0.07
2	49/70 (70.0%)	416/588 (70.7%)	0.90
3	19/70 (27.1%)	149/588 (25.3%)	0.74
Timi Flow Grade			0.15
3	47 (67%)	317 (54%)	
2	8 (11%)	114 (19%)	
1	7 (10%)	90 (15%)	
0	8 (11%)	67 (11%)	
Multivessel	22/70 (31%)	101/588 (17%)	0.004
Multisegmented	23/70 (33%)	120/588 (20%)	0.02

Variables obtained from adjudicated coronary angiograms of n=658 participants in this dataset comparing pregnancy associated spontaneous coronary artery dissection (P-SCAD) vs non pregnancy related SCAD (NP-SCAD) individuals within the international spontaneous coronary artery dissection (iSCAD) registry. Data is presented as n or n/n(data available) (%) or median (IQR) depending on categorical or continuous variables, respectively. LAD: left anterior descending, LCx: left circumflex, RCA: right coronary artery.

© 2026 Koczo A et al. *JAMA Cardiology*.

eTable 2. Mental Health Questionnaires

Baseline Mental Health Variables	P-SCAD (n=95)	NP-SCAD (n=786)	P-Value
Anxiety symptom severity (GAD-7)<br>GAD-7 scores of probable anxiety	4.0 (1.0, 7.0)<br>14/95 (14.7%)	3.0 (0.0, 6.0)<br>98/786 (12.5%)	0.06
Depression symptom severity (PHQ-8)<br>PHQ-8 scores of probable depression	3.0 (1.0, 7.0)<br><br>9/95 (9.5%)	3.0 (1.0, 7.0)<br><br>125/786 (15.9%)	0.32
PTSD symptom severity (PCL-5)<br>PTSD scores of probable PTSD	9.0 (4.0, 17.0)<br>8/95 (8.4%)	7.0 (2.0, 15.0)<br>125/786 (15.9%)	0.08
Sleep disturbance T-score (PROMIS 6a)<br>Sleep disturbance >1 SD above mean	50.9 (42.5, 54.8)<br>13/94 (13.8%)	50.9 (46.4, 57.3)<br>111/786 (14.1%)	0.10

Validated mental health questionnaires variables comparing women with pregnancy-associated spontaneous coronary artery dissection (P-SCAD) vs non pregnancy related SCAD (NP-SCAD). Data is presented as n or n/n(data available) (%) or median (IQR) depending on categorical or continuous variables (normal vs non-normal distribution), respectively.

© 2026 Koczo A et al. *JAMA Cardiology*.

eTable 3. Comparison of P-SCAD Variables in iSCAD vs. Other Registries

Variables	iSCAD<br>(n=98)	Tweet et al.<br>(n=54)	Havekuk et al.<br>(n=120)	Chan et al.<br>(n=82)
Age (years)	39.2 (6.6)	35 (26-42)	34 (4)	36 (IQR 5)
Self-identified Race<br>-White	74 (76%)	48 (89%)	---	85%
Chronic hypertension	18 (18%)	15 (28%)	5/95 (5%)	15%
Dyslipidemia	4 (4%)	---	9/94 (10%	13%
Diabetes mellitus	1 (1%)	---	4/95 (4%)	5%
Connective Tissue Conditions<br>-FMD<br>-Inherited vasculopathies	27/86 (31%)	11 (42%)	---<br>2/44 (5%)	16% (extra coronary arteriopathy)
**Adverse pregnancy outcomes**<br>-Preeclampsia<br>-Eclampsia<br>-Gestational diabetes	24 (25%)<br>2 (2%)<br>11 (11%)	6 (11%)<br>0 (0%)<br>4 (7%)	8/65 (12%)<br>(preE or E)	---<br>---<br>---
**Reproductive history**				
History of ART<br>-Any history of fertility treatment	25/97 (26%)	15 (28%)	---	---
Gravida<br>>1 pregnancy	88 (87%)	49 (91%)	---	---
Parity<br>>1 child	75 (75%)	43 (80%)	---	---
**SCAD Event**				

© 2026 Koczo A et al. *JAMA Cardiology*.

Presentation				
-Unstable angina	26 (30%)	0 (0%)	---	
-NSTEMI	44 (51%)	23 (43%)	---	
-STEMI	16 (19%)	30 (57%)		40/82 (49%)
Coronary Location				
-Left main	0/70 (0%)	13 (24%)	43 (36%)	---
-Proximal	---			31/82 (38%)
-Multivessel	22/70 (31%)		48(40%)	15/81 (19%)
-Multisegmented	23/70 (33%)	18 (33%)		46/81 (57%)
LVEF at presentation (%)	48.8 (12.1)	52% (11)	40±9	
**Management**				
Medical management only	72/97 (74%)	22 (41%)	33 (28%)	46/82 (56%)
PCI	22/97 (23%)	23 (43%)	44 (36%)	
CABG	3/97 (3%)	14 (26%)	44 (37%)	10/82
Thrombolytics	1/90 (1%)		10 (8%)	
In-hospital death	0 (0%)	0 (0%)	1 (1%)	

Comparison of clinical, reproductive and SCAD-specific variables from this study with other large pregnancy associated spontaneous coronary artery dissection studies.ART: assisted-reproductive technologies, CABG: coronary artery bypass graft, NSTEMI- non-ST elevation MI, STEMI- ST elevation MI.

© 2026 Koczo A et al. *JAMA Cardiology*.
