# Supplementary material for: Pregnancy-Associated Spontaneous Coronary Artery Dissection: A Report of the iSCAD Registry
Source: JAMA Cardiol. 2026 Mar 29;11(6):534–43. doi: 10.1001/jamacardio.2026.1009 (PMC13034164; doi:10.1001/jamacardio.2026.1009)
Supplement: Supplement 2. — Data Sharing Statement. [file jamacardiol-e261009-s002.pdf]

Data Sharing Statement

Koczo. Pregnancy-Associated Spontaneous Coronary Artery Dissection. *JAMA Cardiol*. Published March 29, 2026. doi:10.1001/jamacardio.2026.1009

Data
Data available: No

Additional Information
Explanation for why data not available: Given patient identifiers are part of the dataset, data will not be shared.
